# Moves to age-restricted housing and functional health trajectories among independent living older adults

**DOI:** 10.1093/geroni/igab046.2360

**Published:** 2021-12-17

**Authors:** Noah Webster, Simon Brauer

**Affiliations:** University of Michigan, Ann Arbor, Michigan, United States

## Abstract

Where independent living older adults live has been found to have strong links with disability. For example, older adults living in age-restricted housing contexts (e.g., retirement communities) have been found to have worse functional health compared to those living in non-age-restricted settings. Theories and empirical research demonstrate positive and negative aspects of living in age-restricted housing. Recent availability of population-level longitudinal data with sufficiently large samples has made examination of this heterogeneity possible. In this study we examine whether a move to age-restricted housing is associated with functional health trajectories and whether age at time of move moderates this link. We examine these questions using nine waves of longitudinal data from a representative sample of 8,687 U.S. adults age 65 and older from the National Health and Aging Trends Study. Spline-like growth curve models were estimated to determine the intercept, slope prior to move to age-restricted housing, and slope after the move. We also tested whether these processes are conditional on age at time of move. Results indicate that regardless of age all respondents experienced a decline in functional health following a move to age-restricted housing. However, there is variation in the steepness of this decline by age at time of the move. People who move to age-restricted housing earlier experience a less steep decline in functional health post-move compared to those who move later. Findings suggest moving to age-restricted housing earlier may enable older adults to utilize resources often available in these settings to prevent steep health declines.

